# Supplementary material for: From sequence to enzyme mechanism using multi-label machine learning
Source: BMC Bioinformatics. 2014 May 19;15:150. doi: 10.1186/1471-2105-15-150 (PMC4229970; doi:10.1186/1471-2105-15-150)
Supplement: Additional file 2 — Java code of ml2db. Additional file ml2db_code.tar.gz contains the Java source code to run the multi-label machine learning experiments and save the results to database. The code’s Javadoc is included. [file 1471-2105-15-150-S2.zip › additional file 2/ml2db/utils/doc/uk/ac/standrews/utils/main/webutils/simpledomparser/XmlNode.html]

XmlNode


JavaScript is disabled on your browser.


- Overview
- Package
- Class
- Use
- Tree
- Deprecated
- Index
- Help

- Prev Class
- Next Class

- Frames
- No Frames

- All Classes

- Summary:
- Nested |
- Field |
- Constr |
- Method

- Detail:
- Field |
- Constr |
- Method


uk.ac.standrews.utils.main.webutils.simpledomparser

## Class XmlNode

- java.lang.Object
- - uk.ac.standrews.utils.main.webutils.simpledomparser.XmlNode

- ---

    

  ```
  public class XmlNode
  extends java.lang.Object
  ```

  `SimpleElement` is the only node type for simplified DOM model.
  Adapted from: http://www.devx.com/xml/Article/10114 by Guang Yang

  Version:
  :   12 Dec 2007

  Author:
  :   Luna De Ferrari luna.deferrari-at-ed.ac.uk (adapted from Guang Yang)

- - ### Constructor Summary

    Constructors

    Constructor and Description

    `XmlNode(java.lang.String tagName)`
  - ### Method Summary

    Methods

    | Modifier and Type | Method and Description |
    | --- | --- |
    | `XmlNode` | `addChildNode(XmlNode element)` Adds a child element to the node, and returns the node itself |
    | `java.lang.String` | `getAttributeValue(java.lang.String attributeName)` Get the attribute value for the given attribute name. |
    | `java.util.LinkedList<XmlNode>` | `getChildElements()` public Object[] getChildElements() { return m\_childElements.toArray(); } |
    | `java.lang.String` | `getTagName()` Get the tag E.g. |
    | `java.lang.String` | `getText()` Get text for the tag E.g. |
    | `static XmlNode` | `getXmlTreeFromString(java.lang.String XMLText)` |
    | `static XmlNode` | `getXmlTreeFromUrl(java.lang.String url, boolean gzipped)` get XML tree from URL |
    | `boolean` | `hasChildren()` Whether a SimpleElement node has child nodes. |
    | `java.lang.String` | `nodeHeadToString()` String representation of the node opening tag |
    | `void` | `removeAttribute(java.lang.String name)` remove an attribute by name |
    | `XmlNode` | `removeChildNode(XmlNode node)` Remove a child element |
    | `void` | `setAttribute(java.lang.String name, java.lang.String value)` |
    | `void` | `setTagName(java.lang.String tagName)` |
    | `void` | `setText(java.lang.String text)` |
    | `java.lang.String` | `toString()` Full string representation of node and all child nodes. |

    - ### Methods inherited from class java.lang.Object

      `equals, getClass, hashCode, notify, notifyAll, wait, wait, wait`

- - ### Constructor Detail


    - #### XmlNode

      ```
      public XmlNode(java.lang.String tagName)
      ```
  - ### Method Detail


    - #### getXmlTreeFromString

      ```
      public static XmlNode getXmlTreeFromString(java.lang.String XMLText)
      ```


    - #### getXmlTreeFromUrl

      ```
      public static XmlNode getXmlTreeFromUrl(java.lang.String url,
                              boolean gzipped)
      ```

      get XML tree from URL

      Parameters:
      :   `url` -
      :   `gzipped` - whether the url has to be gunzipped

      Returns:
      :   and XML tree


    - #### addChildNode

      ```
      public XmlNode addChildNode(XmlNode element)
      ```

      Adds a child element to the node, and returns the node itself

      Parameters:
      :   `element` - the child node to be added

      Returns:
      :   the node itself, now containing also the child node


    - #### getAttributeValue

      ```
      public java.lang.String getAttributeValue(java.lang.String attributeName)
      ```

      Get the attribute value for the given attribute name. E.g. for a
      SimpleElement 0<\/Item>
      this.getAttribute("Name") would return "Status"

      Parameters:
      :   `attributeName` - the attribute name

      Returns:
      :   the attribute value for the attribute name.


    - #### getChildElements

      ```
      public java.util.LinkedList<XmlNode> getChildElements()
      ```

      public Object[] getChildElements() { return m\_childElements.toArray(); }


    - #### getTagName

      ```
      public java.lang.String getTagName()
      ```

      Get the tag E.g. for a SimpleElement 0<\/Item> this.getTag() would return "Item"

      Returns:
      :   the XML tag name


    - #### getText

      ```
      public java.lang.String getText()
      ```

      Get text for the tag E.g. for a SimpleElement <Item Name="Status"
      Type="Integer">0</Item> this.getText() would return "0"

      Returns:
      :   the text value in between the tag


    - #### hasChildren

      ```
      public boolean hasChildren()
      ```

      Whether a SimpleElement node has child nodes.

      Returns:
      :   true if the SimpleElement has children nodes.


    - #### nodeHeadToString

      ```
      public java.lang.String nodeHeadToString()
      ```

      String representation of the node opening tag

      Returns:


    - #### removeAttribute

      ```
      public void removeAttribute(java.lang.String name)
      ```

      remove an attribute by name

      Parameters:
      :   `name` -


    - #### removeChildNode

      ```
      public XmlNode removeChildNode(XmlNode node)
      ```

      Remove a child element

      Parameters:
      :   `node` - a child node

      Returns:


    - #### setAttribute

      ```
      public void setAttribute(java.lang.String name,
                      java.lang.String value)
      ```


    - #### setTagName

      ```
      public void setTagName(java.lang.String tagName)
      ```


    - #### setText

      ```
      public void setText(java.lang.String text)
      ```


    - #### toString

      ```
      public java.lang.String toString()
      ```

      Full string representation of node and all child nodes.

      **Overrides:**
      :   `toString` in class `java.lang.Object`


- Overview
- Package
- Class
- Use
- Tree
- Deprecated
- Index
- Help

- Prev Class
- Next Class

- Frames
- No Frames

- All Classes

- Summary:
- Nested |
- Field |
- Constr |
- Method

- Detail:
- Field |
- Constr |
- Method
